# Effects of aerobic exercise in the treatment of older adults with chronic musculoskeletal pain: a protocol of a systematic review

**DOI:** 10.1186/s13643-019-1165-7

**Published:** 2019-10-30

**Authors:** Naiane Teixeira Bastos de Oliveira, Irlei dos Santos, Gisela Cristiane Miyamoto, Cristina Maria Nunes Cabral

**Affiliations:** Masters and Doctoral Programs in Physical Therapy, Universidade Cidade de São Paulo, Rua Cesário Galeno, 448/475, Tatuapé, São Paulo, SP 03071-000 Brazil

**Keywords:** Chronic musculoskeletal pain, Aerobic exercise, Older adults, Systematic review

## Abstract

**Background:**

Chronic musculoskeletal pain affects the quality of life of older adults by interfering in their ability to perform activities of daily living. Aerobic exercise programs have been used in the treatment of various health conditions, including musculoskeletal disorders. However, there is still little evidence on the effects of aerobic exercise for the treatment of older adults with chronic musculoskeletal pain. Thus, the objective of this study is to assess the effects of aerobic exercise in improving pain and function of older adults with chronic pain as a consequence of different chronic musculoskeletal conditions.

**Methods:**

The databases to be used in the search are PubMed, EMBASE, CINAHL, PEDro, and Cochrane Central Register of Controlled Trials (CENTRAL)*.* Randomized controlled trials that used aerobic exercise in the treatment of older adults with chronic musculoskeletal pain will be included. Primary outcomes will be pain and function. We will use the PEDro scale to evaluate the methodological quality and statistical description of each included study, and the strength of the recommendations will be summarized using GRADE.

**Discussion:**

The results of this systematic review will provide a synthesis of the current evidence on the effects of aerobic exercise in the treatment of older adults with chronic musculoskeletal pain. In addition, this information can help health professionals in decision-making about the use of aerobic exercise in the treatment of older adults with chronic musculoskeletal pain.

**Ethics and dissemination:**

This systematic review was recorded prospectively, and the results will be part of a doctoral thesis to be published in a peer-reviewed international journal and possibly presented at international conferences.

**Systematic review registration:**

PROSPERO, CRD42019118903.

## Background

Aging is a natural process that includes a dynamic and irreversible decline in physiological function, usually associated with an increase in the manifestation of chronic degenerative diseases [1]. Pain can be characterized as an unpleasant, sensitive, and emotional experience, associated or not with actual or potential tissue damage [2]. With aging, the global prevalence of chronic pain increases, and in 50 to 75% of cases, it may be underdiagnosed or undertreated [3].

Musculoskeletal pain is one of the main types of chronic pain in older adults [4], affecting approximately 50% of community-dwelling older adults [5]. Chronic musculoskeletal pain is one of the main causes of disability in older adults and is associated with difficulties with mobility and daily activities. It affects more women than men and generates high socioeconomic costs [4, 6, 7]. A systematic review indicated that older adults with chronic musculoskeletal pain are less active and may present with disability combined with poor mobility, frailty, depression, cognitive impairment, falls, and poor quality of sleep [4]. Changes in the pain signal associated with aging include a decrease in the integrity and density of cellular elements in the peripheral nervous system, leading to loss of nociceptive function [8, 9]. In the central nervous system, there is a reduction in the neurotransmission paths, affecting the adequate transmission of the pain signal and its neuromodulation [9, 10]. In addition, a systematic review with meta-analysis suggests that older adults tend to have greater intolerance to pain and an increased perception of pain [11].

The treatment of chronic musculoskeletal pain in older adults involves pharmacological and non-pharmacological interventions [12]. Due to the short- and long-term side effects of medication, the non-pharmacological approach has been gaining prominence [9]. Among non-pharmacological interventions, physical exercise is an option, with the objective of preserving the functional independence and quality of life of older adults [9]. Exercise interventions for older adults with chronic musculoskeletal pain should meet the needs of each patient, and should consider their preferences for type and mode of exercise [13].

Regular physical exercise has a protective effect on cardiovascular changes, depressive symptoms, and physical disuse in older adults [14, 15]. In addition, it may limit the development and progression of disabling conditions [15], such as chronic musculoskeletal pain. In the USA, the Centers for Disease Control and Prevention recommend that older adults perform strengthening exercises and aerobic activities to reduce the risk of mortality [16]. Aerobic exercise for 30 to 40 min stimulates the production of endorphins, which bind to opioid receptors in the pain control system of the brain and spinal cord to decrease the perception of pain [17]. To date, only one systematic review has been published [18] with the objective of verifying the effects of walking in patients with chronic musculoskeletal pain. Improvements in pain and short-term function were observed; however, this systematic review did not include other modalities of aerobic exercise and the results are not specific for older adults [18]. Thus, no systematic review has verified the effects of different types of aerobic exercise in the treatment of older adults with chronic musculoskeletal pain. Therefore, the objective of this study is to assess the effect of aerobic exercise on pain and function in older adults with chronic pain caused by different musculoskeletal conditions.

## Methods

### Study design

Systematic review.

### Inclusion criteria

#### Study design

Only published randomized controlled trials assessing the use of aerobic exercise in older adults with chronic pain caused by various musculoskeletal conditions compared to any other type of medical or non-medical intervention or no intervention will be included.

#### Participants

We will include studies that assess older adults aged 65 years or more, that is, all the participants of the study should be aged over 65, from both sexes, and with chronic musculoskeletal pain. Chronic musculoskeletal pain will be defined as any muscle, joint, or tendon pain present for a minimum of 3 months [19]. Studies that assess older adults with chronic pain of non-musculoskeletal origin, such as cancer, will be excluded.

#### Types of intervention and comparison

The investigated intervention will be aerobic exercise used in the treatment of chronic musculoskeletal pain, such as walking, swimming, and cycling, among others. There will be no restrictions in the included studies regarding which professional prescribed the exercise and whether the exercise was supervised or not. Studies included can present the intervention of interest compared to a placebo group, control group with no intervention or minimal intervention (such as waiting list or follow-up booklets), other interventions (medical/pharmacological treatment, physical therapy, yoga, or other exercise modalities such as stabilizing and strengthening exercises) and other types of aerobic exercise.

#### Outcomes

Primary outcomes will be pain intensity (e.g., measured by the Pain Numerical Rating Scale) and function (e.g., measured by the Patient-Specific Functional Scale), assessed by means of questionnaires or specific tests. The secondary outcomes included will be quality of life (e.g., measured by the SF-36 Quality of Life Questionnaire), depression (e.g., measured by the Geriatric Depression Scale), sleep quality (e.g., measured by the Pittsburgh Sleep Quality Index), kinesiophobia (e.g., measured by the Tampa Scale for Kinesiophobia), and adverse effects. The outcomes will be classified into three periods: periods close to 4 weeks will be classified as short term, periods close to 6 months will be medium term, and periods close to 1 year will be long term [20].

### Search procedures and selection of studies

The searches will be performed in the following databases: PubMed, EMBASE, CINAHL, PEDro, and Cochrane Central Register of Controlled Trials (CENTRAL). The search strategy is shown in Additional file 1. Manual searches will also be carried out through the reference list of previous systematic reviews on the topic and of the clinical trials included in this review. Searches will not be restricted by language or date of publication [21, 22]. We plan to finish the search on August 30, 2019.

### Data collection and analysis

#### Selection of studies

The studies will be assessed according to the eligibility criteria, and the selection will be divided into two phases. Initially, two independent reviewers will select the titles of the articles, and in the second phase, the reviewers will read the abstracts and full texts. Any disagreement will be resolved by a third reviewer. In case of doubt regarding the eligibility of an article, the authors may be contacted for clarification.

#### Data extraction and management

The data will be extracted onto an Excel spreadsheet containing information such as authors’ name, place and year of publication, type of chronic musculoskeletal disease, and assessed outcomes. In addition, data on sample characteristics and size, characteristics of interventions performed, instruments used to assess outcomes, results of included studies, and follow-up of the study will also be extracted. The spreadsheet will be pre-tested with two randomized controlled trials similar to those eligible in this review. Two independent reviewers will perform the data extraction, and any disagreements will be resolved by a third reviewer. When data is not available in the manuscripts or if data is unclear, the authors of the studies may be contacted for clarification. All data from questionnaires presented on different scales will be converted to a scale ranging from 0 to 100.

#### Assessment of risk of bias

The assessment for risk of bias and statistical description of the studies will be performed by the PEDro scale, which has good validity and reliability levels, and is strongly correlated with the risk of bias scale from the Cochrane Collaboration [23, 24]. This scale has 11 items: 8 items (items 2–9) refer to methodological quality (random allocation, concealed allocation, baseline similarity, blinding of therapist, blinding of patient, blinding of assessor, appropriate follow-up, and intention to treat analysis) and 2 items (10 and 11) refer to the statistical description (between-group statistical comparison, point measures, and measures of variability) [24]. The first item (eligibility criteria) is not considered in the total score because it is related to external validity [24]. The total PEDro score ranges from 0 to 10 points; the higher the score, the better the methodological quality and statistical description of the article [24]. For studies that are not available in the PEDro database, the PEDro scale will be applied by two independent reviewers and a third reviewer will mediate any disagreements. The studies will be considered as low risk of bias if they have a score equal to or higher than 6 points and as a high risk of bias with a score lower than 6 points [25].

#### Measures of treatment effect

The effects of treatment for continuous outcomes will be reported by determining the effect size for pain intensity and function. If data is sufficient, meta-analyses will be performed using the random-effects model according to the short-, medium-, and long-term follow-up periods [20] to analyze pain intensity and function through the mean difference and 95% confidence intervals. Sensitivity analysis will be conducted to identify the results of the effectiveness between groups when the studies present a high risk of bias. If possible, subgroup analyses will be performed for the musculoskeletal diseases and for age. Meta-analyses will be performed in the Review Manager 5.2.

#### Analysis of heterogeneity

To identify the heterogeneity in the data from the included studies the chi-square test will be used. The magnitude of the heterogeneity will be ascertained by calculating *I*^2^, a measure that ranges from 0 to 100% [24]. An I^2^ above 50% indicates significant heterogeneity and will result in a reduction of one level in the quality of the evidence due to inconsistency [20, 23–25].

#### Synthesis of data

The quality of the evidence will be classified using the GRADE (Grading of Recommendations Assessment, Development, and Evaluation) approach [25]. According to GRADE, evidence quality assessment is performed for each outcome, and the combined available evidence is considered. The quality of evidence is classified into four levels (high, moderate, low, and very low) based on the comprehensive assessment of inconsistency, indirect evidence (not generalizable), inaccuracy, and publication bias. These levels represent confidence in the estimation of the treatment effects presented (Table 1) [26]. The level of evidence and strength of recommendation will be determined by discussion involving all authors. As we expect some degree of heterogeneity, narrative synthesis of the results would be used as needed.

**Table 1:** Level of quality of evidence according to GRADE [26]

GRADE Score	Quality	Interpretation
≤ 1	Very low	Any estimation of effect is highly uncertain
2	Low	Further research is likely to have a significant impact on our confidence in the estimated treatment effect and may change the estimate
3	Moderate	Further research is likely to have a significant impact on our confidence in the estimated treatment effect and may change the estimate
≥ 4	High	It is very unlikely that further research will alter our confidence in the estimated treatment effect

## Discussion

This systematic review aims to summarize the available evidence on the studies that verified the effects of aerobic exercise in improving chronic musculoskeletal pain in older adults. So far, we are unaware of any similar published systematic review. To obtain a high-quality study, we will follow all the recommendations of the Cochrane Handbook of Systematic Reviews. Primary outcomes were chosen taking into account their importance in the assessment of chronic musculoskeletal pain in older adults, so that the results of this review can easily be compared or combined with those of other systematic reviews on the treatment of chronic musculoskeletal pain. The results of this systematic review will inform physical therapists and other health professionals, as well as patients, about the value of an intervention based on aerobic exercise, given that it is an affordable, low-cost intervention commonly used by the general population. In addition, this study can identify gaps in the literature and guide future studies.

### Ethics and dissemination

This study was registered prospectively, and the results will form part of a doctoral thesis and will be published in a peer-reviewed international journal and presented at international conferences.

## Supplementary information

**Additional file 1.** Detailed search strategy.

## Data Availability

Not applicable.

## Abbreviations

CINAHL: Cumulative Index to Nursing and Allied Health Literature
EMBASE: Excerpta Medica dataBASE
GRADE: Grading of Recommendations Assessment, Development, and Evaluation
PEDro: Physiotherapy Evidence Database
PUBMED: National Library of Medicine
