# Supplementary material for: Effects of aerobic exercise in the treatment of older adults with chronic musculoskeletal pain: a protocol of a systematic review
Source: Syst Rev. 2019 Oct 30;8:250. doi: 10.1186/s13643-019-1165-7 (PMC6820912; doi:10.1186/s13643-019-1165-7)
Supplement: Supplementary file 1 — Additional file 1. Detailed search strategy. [file 13643_2019_1165_MOESM1_ESM.docx]

**Additional file 1**

**Search strategy for PUBMED**

***Part A: Intervention***

#1 "physical exercises"[tiab] OR "physical exercise"[tiab] OR "physical activities"[tiab] OR "physical activity"[tiab] OR "physical recreation"[tiab] OR exercise[Mesh] OR mountaineering[tiab] OR mountaineering[Mesh] OR golf[tiab] OR golf[Mesh] OR gymnastics[tiab] OR gymnastic[tiab] OR gymnastic[Mesh] OR "Weight Lifting"[tiab] OR "Weight Bearing"[tiab] OR strengthening[tiab] OR "Resistance Training"[Mesh] OR dancing[tiab] OR dancing[Mesh] OR skating[tiab] OR skating[Mesh] OR jogging[tiab] OR jogging[Mesh] OR cycling[tiab] OR bicycling[tiab] OR bicycling[Mesh] OR pedal[tiab] OR rowing[tiab] OR walking[tiab] OR walking[Mesh] OR running[tiab] OR running[Mesh] OR swimming[tiab] OR swimming[Mesh] OR "physical activities"[tiab] OR "physical activity"[tiab] OR "physical activities"[Mesh] OR "acute exercise"[tiab] OR "acute exercises"[tiab] OR "isometric exercises"[tiab] OR "isometric exercise"[tiab] OR "aerobic exercise"[tiab] OR "aerobic exercises"[tiab] OR "exercise training"[tiab] OR "exercise trainings"[tiab] OR "exercise therapies"[tiab] OR "exercise therapy"[tiab] OR "exercise therapy"[Mesh] OR "rehabilitation exercise"[tiab] OR "rehabilitation exercises"[tiab] OR "remedial exercise"[tiab] OR "remedial exercises"[tiab] OR "exercise movement techniques"[Mesh] OR "exercise movement techniques"[tiab] OR Pilates[tiab] OR "physical exertions"[tiab] OR "physical exertion"[tiab] OR "physical effort"[tiab] OR "physical efforts"[tiab] OR sports[Mesh] OR sport[tiab] OR sports[tiab] OR "cardiorespiratory fitness"[tiab] OR "cardiorespiratory fitness"[Mesh] OR athletics[tiab] OR athletic[tiab] OR aquarobics[tiab] OR slimnastics[tiab] OR warm-up[tiab] OR workout[tiab] OR endurance[tiab] OR exercises[tiab] OR "exercise tolerance"[tiab] OR "aerobic capacity"[tiab] OR "physical capacity"[tiab] OR "physical endurance"[tiab] OR "leisure activity"[tiab] OR "leisure activities"[tiab]

***Part B: Study design***

#2 "Randomized Controlled Trials as Topic"[Mesh] OR "Controlled Clinical Trials as Topic"[Mesh] OR "Clinical Trials as Topic"[Mesh] OR "Randomized Controlled Trial"[Publication Type] OR "Controlled Clinical Trial"[Publication Type] OR "Clinical Trial" [Publication Type] OR "Random Allocation"[Mesh] OR "Single-Blind Method"[Mesh] OR "Double-Blind Method"[Mesh] OR "Research Design"[Mesh] OR "Comparative Study" [Publication Type] OR "Evaluation Studies" [Publication Type] OR "Evaluation Studies as Topic"[Mesh] OR "Drug Therapy"[Mesh] OR "drug therapy"[Subheading] OR "Follow-Up Studies"[Mesh] OR "Cross-Over Studies"[Mesh] OR "Prospective Studies"[Mesh] OR "Clinical Study" [Publication Type] OR "Controlled Before-After Studies"[Mesh] OR "Multicenter Studies as Topic"[Mesh] OR "Multicenter Study" [Publication Type] OR "Placebos"[Mesh] OR Random*[tiab] OR "latin square"[tiab] OR pragmatic trial*[tiab] OR clinical article*[tiab] OR placebo*[tiab] OR ((singl*[tiab] OR doubl*[tiab] OR trebl*[tiab] OR tripl*[tiab]) AND (mask*[tiab] OR blind*[tiab] OR dumm*[tiab])) OR RCT*[tiab] OR ((comparative[tiab] OR control*[tiab] OR clinical[tiab] OR prospectiv*[tiab]) AND (study[tiab] OR studies[tiab] OR trial*[tiab])) OR volunteer*[tiab] OR "Cross-Over"[tiab] OR crossover[tiab] OR allocat*[tiab] OR assign*[tiab] OR factorial[tiab]

***Part C: Participants***

*Elderly*

#3 geriatric*[tiab] OR elder*[tiab] OR old-age*[tiab] OR pensioner*[tiab] OR aging[tiab] OR aged[tiab] OR senior*[tiab] OR old*[tiab] OR adult*[tiab] OR citizen*[tiab] OR resident[tiab] OR residents[tiab] OR "population* elderly"[tiab] OR geriatric*[tiab] OR "long-term care"[tiab] OR "older people"[tiab] OR postmenopausal[tiab] OR "community dwelling"[tiab] OR "middle age"[tiab] OR "middle aged"[tiab]

*Chronic musculoskeletal pain*

#4 "chronic pain"[tiab] OR "widespread pain"[tiab] OR fibromyalgia[tiab] OR "back pain"[tiab] OR "lumbar pain"[tiab] OR "lumbo* pain"[tiab] OR "spine pain"[tiab] OR "spinal pain"[tiab] OR "neck pain"[tiab] OR "cervical pain"[tiab] OR whip-lash[tiab] OR whiplash[tiab] OR knee*[tiab] OR "hip pain" OR "shoulder* pain"[tiab] OR "foot pain"[tiab] OR "feet pain"[tiab] OR "ankle* pain"[tiab] OR "elbow* pain"[tiab] OR "musculo pain"[tiab] OR "muscular pain"[tiab] OR "musculoskel* pain"[tiab] OR "complex regional pain syndrome"[tiab] OR "regional pain"[tiab] OR "neuropath* pain"[tiab] OR "radicular pain"[tiab] OR "non malignant pain"[tiab] OR "non cancer pain"[tiab] OR arthralg*[tiab] OR causalg*[tiab] OR myalg*[tiab] OR myofasc*[tiab] OR polymyalg*[tiab] OR arthralgia[tiab] OR neuralgia*[tiab] OR "musculoskeletal diseases"[tiab] OR "musculoskeletal system"[tiab] OR muscular[tiab] OR musculo*[tiab] OR osteoarthrit*[tiab] OR spondylitis[tiab] OR spondylosis[tiab] OR osteitis[tiab] OR osteochondritis[tiab] OR arthropathy[tiab] OR neurogenic[tiab] OR bursitis[tiab] OR lordosis[tiab] OR lumbago[tiab] OR cervicogenic[tiab] OR sciatic[tiab] OR dyskinesis[tiab] OR tendinitis[tiab] OR allodynia[tiab] OR hyperalgesia[tiab] OR sacroiliac[tiab] OR subluxation[tiab]

***Part D: Final connections***

#5 ((#1 AND #2 AND #3 AND #4) NOT (animals[mh] NOT humans[mh]))

**Search strategy for EMBASE**

***Part A: Intervention***

#1 "physical exercises":ti,ab OR "physical exercise":ti,ab OR "physical activities":ti,ab OR "physical activity":ti,ab OR "physical recreation":ti,ab OR 'exercise'/exp OR mountaineering:ti,ab OR 'mountaineering'/exp OR golf:ti,ab OR 'golf'/exp OR gymnastics:ti,ab OR gymnastic:ti,ab OR 'gymnastic'/exp OR "Weight Lifting":ti,ab OR "Weight Bearing":ti,ab OR strengthening:ti,ab OR 'Resistance Training'/exp OR dancing:ti,ab OR 'dancing'/exp OR skating:ti,ab OR 'skating'/exp OR jogging:ti,ab OR 'jogging'/exp OR cycling:ti,ab OR bicycling:ti,ab OR 'bicycling'/exp OR pedal:ti,ab OR rowing:ti,ab OR walking:ti,ab OR 'walking'/exp OR running:ti,ab OR 'running'/exp OR swimming:ti,ab OR 'swimming'/exp OR "physical activities":ti,ab OR "physical activity":ti,ab OR 'physical activities'/exp OR "acute exercise":ti,ab OR "acute exercises":ti,ab OR "isometric exercises":ti,ab OR "isometric exercise":ti,ab OR "aerobic exercise":ti,ab OR "aerobic exercises":ti,ab OR "exercise training":ti,ab OR "exercise trainings":ti,ab OR "exercise therapies":ti,ab OR "exercise therapy":ti,ab OR 'exercise therapy'/exp OR "rehabilitation exercise":ti,ab OR "rehabilitation exercises":ti,ab OR "remedial exercise":ti,ab OR "remedial exercises":ti,ab OR 'exercise movement techniques'/exp OR "exercise movement techniques":ti,ab OR Pilates:ti,ab OR "physical exertions":ti,ab OR "physical exertion":ti,ab OR "physical effort":ti,ab OR "physical efforts":ti,ab OR 'sports'/exp OR sport:ti,ab OR sports:ti,ab OR "cardiorespiratory fitness":ti,ab OR 'cardiorespiratory fitness'/exp OR athletics:ti,ab OR athletic:ti,ab OR aquarobics:ti,ab OR slimnastics:ti,ab OR warm-up:ti,ab OR workout:ti,ab OR endurance:ti,ab OR exercises:ti,ab OR "exercise tolerance":ti,ab OR "aerobic capacity":ti,ab OR "physical capacity":ti,ab OR "physical endurance":ti,ab OR "leisure activity":ti,ab OR "leisure activities":ti,ab

***Part B: Study design***

#2 'controlled clinical trial'/exp OR 'controlled clinical trial (topic)'/exp OR 'randomized controlled trial'/exp OR 'clinical article'/exp OR 'clinical study'/exp OR 'controlled study'/de OR 'major clinical study'/exp OR 'triple blind procedure'/exp OR 'methodology'/de OR 'comparative study'/de OR 'evaluation study'/exp OR 'follow up'/exp OR 'randomization'/exp OR 'single blind procedure'/exp OR 'double blind procedure'/exp OR 'drug therapy'/exp OR 'crossover procedure'/exp OR 'prospective study'/exp OR 'epidemiology'/de OR 'multicenter study (topic)'/exp OR 'placebo'/exp OR random*:ti,ab OR 'latin square':ti,ab OR 'pragmatic trial*':ti,ab OR 'clinical article*':ti,ab OR placebo*:ti,ab OR (singl*:ti,ab OR doubl*:ti,ab OR trebl*:ti,ab OR tripl*:ti,ab AND (mask*:ti,ab OR blind*:ti,ab OR dumm*:ti,ab)) OR rct*:ti,ab OR (comparative:ti,ab OR control*:ti,ab OR clinical:ti,ab OR prospectiv*:ti,ab AND (study:ti,ab OR studies:ti,ab OR trial*:ti,ab)) OR volunteer*:ti,ab OR 'cross-over':ti,ab OR crossover:ti,ab OR allocat*:ti,ab OR assign*:ti,ab OR factorial:ti,ab

***Part C: Participants***

Elderly

#3 geriatric*:ti,ab OR elder*:ti,ab OR old-age*:ti,ab OR pensioner*:ti,ab OR aging:ti,ab OR aged:ti,ab OR senior*:ti,ab OR old*:ti,ab OR adult*:ti,ab OR citizen*:ti,ab OR resident:ti,ab OR residents:ti,ab OR "population* elderly":ti,ab OR geriatric*:ti,ab OR "long-term care":ti,ab OR "older people":ti,ab OR postmenopausal:ti,ab OR "community dwelling":ti,ab OR "middle age":ti,ab OR "middle aged":ti,ab

*Chronic musculoskeletal pain*

#4 "chronic pain":ti,ab OR "widespread pain":ti,ab OR fibromyalgia:ti,ab OR "back pain":ti,ab OR "lumbar pain":ti,ab OR "lumbo* pain":ti,ab OR "spine pain":ti,ab OR "spinal pain":ti,ab OR "neck pain":ti,ab OR "cervical pain":ti,ab OR whip-lash:ti,ab OR whiplash:ti,ab OR knee*:ti,ab OR "hip pain" OR "shoulder* pain":ti,ab OR "foot pain":ti,ab OR "feet pain":ti,ab OR "ankle* pain":ti,ab OR "elbow* pain":ti,ab OR "musculo pain":ti,ab OR "muscular pain":ti,ab OR "musculoskel* pain":ti,ab OR "complex regional pain syndrome":ti,ab OR "regional pain":ti,ab OR "neuropath* pain":ti,ab OR "radicular pain":ti,ab OR "non malignant pain":ti,ab OR "non cancer pain":ti,ab OR arthralg*:ti,ab OR causalg*:ti,ab OR myalg*:ti,ab OR myofasc*:ti,ab OR polymyalg*:ti,ab OR arthralgia:ti,ab OR neuralgia*:ti,ab OR "musculoskeletal diseases":ti,ab OR "musculoskeletal system":ti,ab OR muscular:ti,ab OR musculo*:ti,ab OR osteoarthrit*:ti,ab OR spondylitis:ti,ab OR spondylosis:ti,ab OR osteitis:ti,ab OR osteochondritis:ti,ab OR arthropathy:ti,ab OR neurogenic:ti,ab OR bursitis:ti,ab OR lordosis:ti,ab OR lumbago:ti,ab OR cervicogenic:ti,ab OR sciatic:ti,ab OR dyskinesis:ti,ab OR tendinitis:ti,ab OR allodynia:ti,ab OR hyperalgesia:ti,ab OR sacroiliac:ti,ab OR subluxation:ti,ab

***Part D: Final connections***

#5 #1 AND #2 AND #3 AND #4 NOT ([animals]/lim NOT [humans]/lim

***Search strategy for Cochrane Central Register of Controlled Trials***

**Part A: Intervention**

#1 "physical exercises":ti,ab OR "physical exercise":ti,ab OR "physical activities":ti,ab OR "physical activity":ti,ab OR "physical recreation":ti,ab OR [mh exercise] OR mountaineering:ti,ab OR [mh mountaineering] OR golf:ti,ab OR [mh golf] OR gymnastics:ti,ab OR gymnastic:ti,ab OR [mh gymnastic] OR "Weight Lifting":ti,ab OR "Weight Bearing":ti,ab OR strengthening:ti,ab OR [mh "Resistance Training"] OR dancing:ti,ab OR [mh dancing] OR skating:ti,ab OR [mh skating] OR jogging:ti,ab OR [mh jogging] OR cycling:ti,ab OR bicycling:ti,ab OR [mh bicycling] OR pedal:ti,ab OR rowing:ti,ab OR walking:ti,ab OR [mh walking] OR running:ti,ab OR [mh running] OR swimming:ti,ab OR [mh swimming] OR "physical activities":ti,ab OR "physical activity":ti,ab OR [mh "physical activities"] OR "acute exercise":ti,ab OR "acute exercises":ti,ab OR "isometric exercises":ti,ab OR "isometric exercise":ti,ab OR "aerobic exercise":ti,ab OR "aerobic exercises":ti,ab OR "exercise training":ti,ab OR "exercise trainings":ti,ab OR "exercise therapies":ti,ab OR "exercise therapy":ti,ab OR [mh "exercise therapy"] OR "rehabilitation exercise":ti,ab OR "rehabilitation exercises":ti,ab OR "remedial exercise":ti,ab OR "remedial exercises":ti,ab OR [mh "exercise movement techniques"] OR "exercise movement techniques":ti,ab OR Pilates:ti,ab OR "physical exertions":ti,ab OR "physical exertion":ti,ab OR "physical effort":ti,ab OR "physical efforts":ti,ab OR [mh sports] OR sport:ti,ab OR sports:ti,ab OR "cardiorespiratory fitness":ti,ab OR [mh "cardiorespiratory fitness"] OR athletics:ti,ab OR athletic:ti,ab OR aquarobics:ti,ab OR slimnastics:ti,ab OR warm-up:ti,ab OR workout:ti,ab OR endurance:ti,ab OR exercises:ti,ab OR "exercise tolerance":ti,ab OR "aerobic capacity":ti,ab OR "physical capacity":ti,ab OR "physical endurance":ti,ab OR "leisure activity":ti,ab OR "leisure activities":ti,ab

***Part B: Participants***

*Elderly*

#2 geriatric*:ti,ab OR elder*:ti,ab OR old-age*:ti,ab OR pensioner*:ti,ab OR aging:ti,ab OR aged:ti,ab OR senior*:ti,ab OR old*:ti,ab OR adult*:ti,ab OR citizen*:ti,ab OR resident:ti,ab OR residents:ti,ab OR "population* elderly":ti,ab OR geriatric*:ti,ab OR "long-term care":ti,ab OR "older people":ti,ab OR postmenopausal:ti,ab OR "community dwelling":ti,ab OR "middle age":ti,ab OR "middle aged":ti,ab

*Chronic musculoskeletal pain*

#3 "chronic pain":ti,ab OR "widespread pain":ti,ab OR fibromyalgia:ti,ab OR "back pain":ti,ab OR "lumbar pain":ti,ab OR "lumbo* pain":ti,ab OR "spine pain":ti,ab OR "spinal pain":ti,ab OR "neck pain":ti,ab OR "cervical pain":ti,ab OR whip-lash:ti,ab OR whiplash:ti,ab OR knee*:ti,ab OR "hip pain" OR "shoulder* pain":ti,ab OR "foot pain":ti,ab OR "feet pain":ti,ab OR "ankle* pain":ti,ab OR "elbow* pain":ti,ab OR "musculo pain":ti,ab OR "muscular pain":ti,ab OR "musculoskel* pain":ti,ab OR "complex regional pain syndrome":ti,ab OR "regional pain":ti,ab OR "neuropath* pain":ti,ab OR "radicular pain":ti,ab OR "non malignant pain":ti,ab OR "non cancer pain":ti,ab OR arthralg*:ti,ab OR causalg*:ti,ab OR myalg*:ti,ab OR myofasc*:ti,ab OR polymyalg*:ti,ab OR arthralgia:ti,ab OR neuralgia*:ti,ab OR "musculoskeletal diseases":ti,ab OR "musculoskeletal system":ti,ab OR muscular:ti,ab OR musculo*:ti,ab OR osteoarthrit*:ti,ab OR spondylitis:ti,ab OR spondylosis:ti,ab OR osteitis:ti,ab OR osteochondritis:ti,ab OR arthropathy:ti,ab OR neurogenic:ti,ab OR bursitis:ti,ab OR lordosis:ti,ab OR lumbago:ti,ab OR cervicogenic:ti,ab OR sciatic:ti,ab OR dyskinesis:ti,ab OR tendinitis:ti,ab OR allodynia:ti,ab OR hyperalgesia:ti,ab OR sacroiliac:ti,ab OR subluxation:ti,ab

***Part C: Final connections***

#4 #1 AND #2 AND #3

**Search strategy for CINAHL**

***Part A: Intervention***

S1 TI "physical exercises" OR AB "physical exercises" OR TI "physical exercise" OR AB "physical exercise" OR TI "physical activities" OR AB "physical activities" OR TI "physical activity" OR AB "physical activity" OR TI "physical recreation" OR AB "physical recreation" OR (MH "exercise+") OR TI mountaineering OR AB mountaineering OR (MH "mountaineering+") OR TI golf OR AB golf OR (MH "golf+") OR TI gymnastics OR AB gymnastics OR TI gymnastic OR AB gymnastic OR (MH "gymnastic+") OR TI "Weight Lifting" OR AB "Weight Lifting" OR TI "Weight Bearing" OR AB "Weight Bearing" OR TI strengthening OR AB strengthening OR (MH "Resistance Training+") OR TI dancing OR AB dancing OR (MH "dancing+") OR TI skating OR AB skating OR (MH "skating+") OR TI jogging OR AB jogging OR (MH "jogging+") OR TI cycling OR AB cycling OR TI bicycling OR AB bicycling OR (MH "bicycling+") OR TI pedal OR AB pedal OR TI rowing OR AB rowing OR TI walking OR AB walking OR (MH "walking+") OR TI running OR AB running OR (MH "running+") OR TI swimming OR AB swimming OR (MH "swimming+") OR TI "physical activities" OR AB "physical activities" OR TI "physical activity" OR AB "physical activity" OR (MH "physical activities+") OR TI "acute exercise" OR AB "acute exercise" OR TI "acute exercises" OR AB "acute exercises" OR TI "isometric exercises" OR AB "isometric exercises" OR TI "isometric exercise" OR AB "isometric exercise" OR TI "aerobic exercise" OR AB "aerobic exercise" OR TI "aerobic exercises" OR AB "aerobic exercises" OR TI "exercise training" OR AB "exercise training" OR TI "exercise trainings" OR AB "exercise trainings" OR TI "exercise therapies" OR AB "exercise therapies" OR TI "exercise therapy" OR AB "exercise therapy" OR (MH "exercise therapy+") OR TI "rehabilitation exercise" OR AB "rehabilitation exercise" OR TI "rehabilitation exercises" OR AB "rehabilitation exercises" OR TI "remedial exercise" OR AB "remedial exercise" OR TI "remedial exercises" OR AB "remedial exercises" OR (MH "exercise movement techniques+") OR TI "exercise movement techniques" OR AB "exercise movement techniques" OR TI Pilates OR AB Pilates OR TI "physical exertions" OR AB "physical exertions" OR TI "physical exertion" OR AB "physical exertion" OR TI "physical effort" OR AB "physical effort" OR TI "physical efforts" OR AB "physical efforts" OR (MH "sports+") OR TI sport OR AB sport OR TI sports OR AB sports OR TI "cardiorespiratory fitness" OR AB "cardiorespiratory fitness" OR (MH "cardiorespiratory fitness+") OR TI athletics OR AB athletics OR TI athletic OR AB athletic OR TI aquarobics OR AB aquarobics OR TI slimnastics OR AB slimnastics OR TI warm-up OR AB warm-up OR TI workout OR AB workout OR TI endurance OR AB endurance OR TI exercises OR AB exercises OR TI "exercise tolerance" OR AB "exercise tolerance" OR TI "aerobic capacity" OR AB "aerobic capacity" OR TI "physical capacity" OR AB "physical capacity" OR TI "physical endurance" OR AB "physical endurance" OR TI "leisure activity" OR AB "leisure activity" OR TI "leisure activities" OR AB "leisure activities"

***Part B: Study design***

S2 (((MH “Clinical Trials+”) OR (“randomi?ed controlled trial*”) OR (clinical W3 trial) OR (double-blind) OR (single-blind) OR (triple-blind)) OR ((MH “Placebo Effect”) OR (MH “Placebos”) OR (“placebo*”) OR (random*)) OR ((MH “Random Sample+”) OR (MH “Study Design+”) OR (“latin square”) OR (MH “Comparative Studies”) OR (MH “Evaluation Research+”) OR (MH “Prospective Studies+”)) OR (follow-up stud*) OR (followup stud*) OR (control*) OR (prospectiv*) OR (volunteer*)) NOT (MH “Animals”)

***Part C: Participants***

*Elderly*

S3 TI geriatric* OR AB geriatric* OR TI elder* OR AB elder* OR TI old-age* OR AB old-age* OR TI pensioner* OR AB pensioner* OR TI aging OR AB aging OR TI aged OR AB aged OR TI senior* OR AB senior* OR TI old* OR AB old* OR TI adult* OR AB adult* OR TI citizen* OR AB citizen* OR TI resident OR AB resident OR TI residents OR AB residents OR TI "population* elderly" OR AB "population* elderly" OR TI geriatric* OR AB geriatric* OR TI "long-term care" OR AB "long-term care" OR TI "older people" OR AB "older people" OR TI postmenopausal OR AB postmenopausal OR TI "community dwelling" OR AB "community dwelling" OR TI "middle age" OR AB "middle age" OR TI "middle aged" OR AB "middle aged"

*Chronic musculoskeletal pain*

S4 TI "chronic pain" OR AB "chronic pain" OR TI "widespread pain" OR AB "widespread pain" OR TI fibromyalgia OR AB fibromyalgia OR TI "back pain" OR AB "back pain" OR TI "lumbar pain" OR AB "lumbar pain" OR TI "lumbo* pain" OR AB "lumbo* pain" OR TI "spine pain" OR AB "spine pain" OR TI "spinal pain" OR AB "spinal pain" OR TI "neck pain" OR AB "neck pain" OR TI "cervical pain" OR AB "cervical pain" OR TI whip-lash OR AB whip-lash OR TI whiplash OR AB whiplash OR TI knee* OR AB knee* OR "hip pain" OR TI "shoulder* pain" OR AB "shoulder* pain" OR TI "foot pain" OR AB "foot pain" OR TI "feet pain" OR AB "feet pain" OR TI "ankle* pain" OR AB "ankle* pain" OR TI "elbow* pain" OR AB "elbow* pain" OR TI "musculo pain" OR AB "musculo pain" OR TI "muscular pain" OR AB "muscular pain" OR TI "musculoskel* pain" OR AB "musculoskel* pain" OR TI "complex regional pain syndrome" OR AB "complex regional pain syndrome" OR TI "regional pain" OR AB "regional pain" OR TI "neuropath* pain" OR AB "neuropath* pain" OR TI "radicular pain" OR AB "radicular pain" OR TI "non malignant pain" OR AB "non malignant pain" OR TI "non cancer pain" OR AB "non cancer pain" OR TI arthralg* OR AB arthralg* OR TI causalg* OR AB causalg* OR TI myalg* OR AB myalg* OR TI myofasc* OR AB myofasc* OR TI polymyalg* OR AB polymyalg* OR TI arthralgia OR AB arthralgia OR TI neuralgia* OR AB neuralgia* OR TI "musculoskeletal diseases" OR AB "musculoskeletal diseases" OR TI "musculoskeletal system" OR AB "musculoskeletal system" OR TI muscular OR AB muscular OR TI musculo* OR AB musculo* OR TI osteoarthrit* OR AB osteoarthrit* OR TI spondylitis OR AB spondylitis OR TI spondylosis OR AB spondylosis OR TI osteitis OR AB osteitis OR TI osteochondritis OR AB osteochondritis OR TI arthropathy OR AB arthropathy OR TI neurogenic OR AB neurogenic OR TI bursitis OR AB bursitis OR TI lordosis OR AB lordosis OR TI lumbago OR AB lumbago OR TI cervicogenic OR AB cervicogenic OR TI sciatic OR AB sciatic OR TI dyskinesis OR AB dyskinesis OR TI tendinitis OR AB tendinitis OR TI allodynia OR AB allodynia OR TI hyperalgesia OR AB hyperalgesia OR TI sacroiliac OR AB sacroiliac OR TI subluxation OR AB subluxation

***Part D: Final connections***

S5 S1 AND S2 AND S3 AND S4

**Search strategy for PEDro**

exercise AND elderly AND chronic musculoskeletal pain
